# Can Agricultural Practices in Strawberry Fields Induce Plant–Nematode Interaction towards *Meloidogyne*-Suppressive Soils?

**DOI:** 10.3390/life12101572

**Published:** 2022-10-10

**Authors:** Mostafa M. A. Hammam, Hassan Abd-El-Khair, Wafaa M. A. El-Nagdi, Mahfouz M. M. Abd-Elgawad

**Affiliations:** Plant Pathology Department, National Research Centre, Dokki, Giza 12622, Egypt

**Keywords:** *Meloidogyne*, suppressive soil, biocontrol, soil microorganisms, strawberry

## Abstract

The importance of benign approaches to manage the root-knot nematodes (RKNs, *Meloidogyne* spp.) in strawberry farms has become more evident with increasing strawberry production and export in Egypt. Therefore, data accumulated on biosolarization and soil amendments to favor beneficial microorganisms and maximize their impact on RKN management are built on a robust historical research foundation and should be exploited. We examined RKN population levels/parameters in three strawberry export governorates, six farms per governorate, to characterize the exact production practices that are responsible for RKN-suppressive soils. All selected farms enjoyed soil biodisinfestation resulting from incorporating organic amendments followed by a plastic cover to suppress soil pathogens. Various safe and inexpensive agricultural practices in the El-Ismailia and El-Beheira governorates were compared to the toxic and expensive fumigants that could eliminate RKNs in the Al-Qalyubia governorate. Two farms at El-Ismailia were of special interest as they ultimately showed almost zero counts of RKNs. The two farms were characterized by incorporating cow manure [containing 0.65% total nitrogen, 21.2 carbon to nitrogen (C/N) ratio] and poultry manure (0.72% total nitrogen, 20.1 C/N ratio) followed by soil solarization via transparent, 80-µm thick plastic covers for 60–65 summer days as pre-strawberry cultivation practices, and similar covers were used after transplanting. Typically, the longer the pre-plant soil solarization period with thicker transparent plastic covers, the better it could suppress the RKN population densities in the tested farms. Their soils were characterized by relatively high pH and low electrical conductivity. The significant development in biocontrol genera/species abundance and frequency could explain the lower (*p* < 0.0001) RKN population levels inhabiting the farms of El-Ismailia than the El-Beheira governorate. These factors could provide the first approximation of key practices and factors that could collectively contribute to distinguishing and exploiting soil suppressiveness against RKNs. We discussed edaphic properties and production practices that could modulate populations of natural RKN antagonists for sustainable strawberry cultivation.

## 1. Introduction

As a specialty and export crop in Egypt, strawberry (*Fragaria* × *ananassa* Duchesne ex Rozier) acreage has been increasing [1]; its acreage has currently increased to approximately 13,635 hectares and fresh strawberry exports from Egypt were raised from nearly 17,500 metric tons in 2010 to more than 35,000 metric tons in 2020 [2]. As strawberry is vegetatively propagated, its high early yields promote fresh strawberry exportation to commence early and continue until the end of the season (mostly in June) [2]. Geographic location, fertile soils, cheap labor, and Mediterranean climate are quite favorable for strawberry growing. These factors reflect the importance of increasing strawberry production via safe methods to manage strawberry pests and secure excellent local consumption and export. Therefore, the significance of benign pesticides and cautious techniques to manage root-knot nematodes (RKNs, *Meloidogyne* spp.) infecting strawberry has become more evident. The RKNs can infest strawberry fields as a single [3,4] or two [5] species, but sometimes as a mixture of *M*. *incognita*, *M*. *javanica*, and *M*. *arenaria* in Egypt [1,6]. Among plant-parasitic nematode (PPN) populations in Egyptian strawberry fields, RKNs have the highest frequency of occurrence and prominence value [6]. Severely *Meloidogyne*-infected plants have reduced root mass with a disordered vascular system in the RKN-galled roots. Thus, the roots are badly hampered in their major tasks of uptake and conveyance of water and nutrients. Meanwhile, berry plants do not duly flower but produce poor quality fruits. Moreover, infected plants are very susceptible to drought damage and less able to compete with other stresses, such as weeds [1]. Egyptian production loss in strawberry yield brought about by PPNs was 33,040.5 metric tons (12%) based on 2011–2012 figures; RKNs have the lion’s share in causing such a loss [7]. The RKN problem on strawberry is so devastating that nematode control is recommended when only one individual per 100 cm^3^ of soil is detected as a pre-plant population density in Florida, USA [8].

Minimizing pesticide residues is required to comply with maximum residue limits for strawberry export. Contrary to chemicals, indigenous biological control agents (BCAs) and their exploitation tactics rank highly as benign alternative control options within integrated pest management (IPM) strategies [9,10]. As RKN-second-stage juveniles (J2) search for plant roots to infect after hatching to complete their life cycle within the roots, these J2 usually migrate through soil. Hence, a slice of their life cycle, namely during both the migration in soil and the frequently exposed egg masses on the root surface, may interact with microorganisms and biocontrol agents (BCAs) in the rhizosphere soil. Yet, some researchers may advocate that it is preferable to study this interaction when these BCAs are either intimately associated with/attached to the nematodes [11] or inside plant roots [12], but not in the soil. Valid as they are, attachment alone does not confirm BCA infection and effectiveness. Although *Pasteuria* spp. are generally known as BCAs, some of these species can sometimes only attach to, but not infect, the nematodes [13]. This trait of attaching to but not infecting the nematodes considerably degrades their value as biocontrol organisms [14]. Studying BCAs inside plant roots may be conducted for a specific target, e.g., examining the induction of plant defense responses by a BCA [12,15]. We would defend the importance of our studying BCAs in arable soils too, as they may build up in the rhizosphere soil with significant effects on RKN regulation [9,16]. Furthermore, some BCAs can act via metabolites and do not need to be intimately associated with the nematodes. Indigenous microbial populations can interact with co-existing *Meloidogyne* spp. in the soil in a variety of beneficial/harmful ways to stimulate or inhibit RKN reproduction [17,18,19]. Admittedly, numerous fungal or bacterial species/isolates can antagonize RKNs in soil and roots either directly, by competing for nutrition and space, predation, parasitism, and toxins, or indirectly, by generating host plant resistance [9,20,21]. Little is known about BCA abundance, diversity, distribution, and possible roles in regulating RKNs in strawberry fields.

As biological soil disinfestation refers to the process resulting from incorporating organic amendments followed by a plastic cover to suppress plant pathogens [22], our current study examined farms that met these conditions. We selected strawberry farms that added organic manure/material, plowed and irrigated the soil before covering with plastic sheets, and finally removed the sheets to set the beds for strawberry transplanting. Such farms were chosen because they met the requirements for biodisinfestation [22,23] as eco-biological methods to control PPNs. During biodisinfestation, microbiological activity notably increases and compounds such as phenols, FeS, or organic acids are generated in the treated soil, which contributes to the inactivation or detriment of pathogens. Additionally, the activated microorganisms may manufacture and release enzymes and other metabolites with anti-pathogenic activities into the soil [23]. Although the agronomic practices carried out in the selected farms are the standard ones, a relatively small number of farms deviate from standard traditions.

Given that Egyptian strawberry growers increasingly adopt these agricultural practices but to varying degrees/techniques, we studied the possible consequent variation in soil microorganisms. Our goals are to: (i) estimate the population densities of RKN parameters and changes in their numbers from three to five months after strawberry transplanting in three main governorates for Egyptian strawberry production and export, (ii) assess the abundance and diversity of fungi and bacteria in soils co-occurring with RKNs (with the presence of various BCAs in strawberry farms, it is expected that habitat-dependent convergence of various microbial communities can lead to differential survival and reproduction of RKNs), and (iii) identify abiotic and biotic factors associated with RKN control or nematode-suppressive soils for sustainable strawberry production. Preliminary data [1,6] have indicated the presence of RKN species *M*. *incognita*, *M*. *javanica*, and *M*. *arenaria* along with soil microorganism communities in all the surveyed fields.

## 2. Materials and Methods

### 2.1. Locations and Cultivation of the Selected Fields

Surveys were conducted in six commercial strawberry farms in each of three governorates, Al-Qalyubia, El-Ismailia, and El-Beheira, in Egypt during the October 2019–June 2020 growing season. The farms were located in the central (Al-Qalyubia), eastern (El-Ismailia), and western (El-Beheira) parts of Egypt. Generally, previously known farms naturally infested with RKNs [1,6] were selected for the study. The Al-Qalyubia farms applied toxic chemical nematicides, so we referred to them only for comparison with the farms of El-Ismailia and El-Beheira, which used alternative soil solarization for pest control. For the latter governorates, production practices that may relate to PPN management in the previous crop of strawberry cultivation were recorded and soil properties were analyzed by the Soil Department, National Research Centre (Table 1); the information was obtained from the growers. Otherwise, eighteen farms similarly prepared for strawberry cultivation and production according to El-Shemy et al. [24] were selected because they fulfilled the following agricultural practices. In July and August, the farms were plowed thrice, with harrowing to soften and level the soil following each plow (Table 2). Organic (mostly cow) manure was added before the last plowing, then the soil was covered with polyethylene sheets of plastic. Chemical fertilizers were added before setting plots, beds, and irrigation network (both overhead sprinklers and irrigation tube drippers), and finally, strawberry seedlings were transplanted in wet soil. The pre-plant sheets were used for either solarization (at the El-Ismailia and El-Beheira farms) or fumigation (at the Al-Qalyubia farms). Comparison of the techniques used is presented in Table 2. In only the Al-Qalyubia farms, the fumigants Agrocelhone and methyl bromide (MB) were used as chemical nematicides for comparison (as controls) with solarization in the other strawberry farms. Approximately 1-m-wide beds for strawberry cultivation were raised 45–50 cm higher than the surrounding soil surface, spaced 0.5–0.6 m apart, and equipped with two longitudinal plastic tubes to irrigate (via drippers) four rows of 0.2-m spaced plants, as well as sprinklers to cover the planted area with an overhead (spray/mist) system. Then, the beds were covered (mulched) with polyethylene sheets of plastic; a hole was made for each plant to exit from the plastic. Irrigation via the drippers only continued until the end of the season.

### 2.2. Collecting Samples for Nematode Extraction, Count, and Identification and Soil Analysis

The soil and root samples were collected from a delimited 3-acre area in each farm. As RKNs develop and tend to cause more damage at mid-season, when plants continue to produce fruit and soils are warmer, samples were taken after approximately three and five months of transplanting strawberry, i.e., at the end of January and March. First sampling was considered the initial population density of root-knot nematodes. That is because most Egyptian soils are too cold to support RKN infection and/or reproduction at earlier sampling. Second sampling thereafter was intended to monitor nematode development and reproduction for at least one generation. Four rhizosphere soil and root subsamples from four random plants were taken with a hand trowel (ca 8 cm diameter × 20 cm deep), mixed and composited into a single composite sample of approximately 1 kg (approximately 4 g of fibrous roots/plant or 16 g per sample). Consequently, five composite samples from 20 plants were taken from each strawberry field each time. Soil and root samples were taken from the same points/plants at the two sampling times to compare the means. Each sample was thoroughly mixed, bagged, labeled, and taken to the laboratory in ice box for nematode analyses. From each sample, an aliquot of 200 g soil was processed by sieving and decanting methods for nematode extraction [25]. The numbers of PPNs in soil were counted using Hawksley slide under light microscope. Fibrous roots from each sample were gently washed free of soil and an aliquot of 5 g roots per sample was considered to count nematode galls before cutting roots into approximately 2-cm-long pieces. At each sampling time, these pieces were placed in Petri dishes with distilled water and incubated under laboratory conditions (25 ± 3 °C) for a week to extract and count J2 stage of *Meloidogyne* nematodes [26]. Other 5 g root samples were stained in 0.015% Phloxine B solution for 20 min before removing the residual Phloxine B to count egg masses [27]. Number of eggs per 5 g roots in these samples was determined by shaking excised roots in 1% NaOCI solution for 3 min; the suspension of eggs was then sieved through 200- and 500-mesh sieves with gentle tap water to remove root pieces and debris on the upper sieve and collect the eggs on the lower one [28]. Released eggs were gathered in 50 mL water suspension and number of eggs was counted in each sample using a light microscope (10×) (Labo America, Inc. New York, NY, USA). Roots were stained by lactophenol acid fuchsine method [29] to count the adult females per 5 g roots via stereoscope (6x) (Labo America, Inc. New York, NY, USA). Identification of RKN species previously known as *Meloidogyne incognita*, *M*. *javanica*, and *M*. *arenaria* [1] was examined herein via perineal pattern morphology of twenty RKN females [30] randomly collected at the second sampling time. Additionally, biochemical identification was adopted by the Nematology Section, Egyptian Ministry of Agriculture using six RKN samples. To determine the esterase phenotype, individual RKN females were macerated in 0.1 phosphate extraction buffer (pH 7.4) with 20% sucrose, 2% Triton x-100, and 0.1% bromophenol blue dye. Electrophoresis of macerated individual females was accomplished with an automated apparatus (Phast system Pharmacia, Uppsala, Sweden) on 10 to 15% gradient polyacrylamide gels according to Esbenshade and Triantaphyllou [31]. Esterase phenotypes were determined by staining polyacrylamide gels for esterase activity according to Tomaszewski et al. [32].

### 2.3. Determining Total Microbial Counts in Samples

The total counts for each of the spore-forming bacteria, aerobic bacteria, and fungi in the rhizosphere soil were determined by the plate count technique using the dilution method [26]. Ten grams of each soil sample was separately shaken in 90 mL of sterilized distilled water in a 250 mL flask to give a dilution of 10^−1^. The count of spore-forming bacteria was determined after pasteurization of the dilution of 10^−1^ at 80 °C for 20 min. Then, 1.0 mL of each 10^−3^ to 10^−5^ dilution was separately transferred onto sterilized Petri plates (10 plates for each dilution) and filled with nutrient agar (NA) medium [peptone 5 g, beef extract 3 g, glucose 20 g, agar 15 g in one liter of distilled water, pH 7]. The count of aerobic bacteria was determined by separately adding 1.0 mL of each 10^−5^ to 10^−7^ dilution onto sterilized Petri plates (10 plates for each dilution) and then filled with NA medium. After two days of incubation at 28 °C, the resulting colonies of spore-forming bacteria and aerobic bacteria were recorded. The count of total fungi was determined in 10^−3^ to 10^−4^ dilution using Martin medium; glucose 10 g, peptone 5 g, KH_2_PO_4_ 1 g, MgSO_4_ 0.5 g, rose bengal 30 μg, agar 15 g in one liter of distilled water [33]. Plates were incubated at 25 ± 2 °C for five days and the resulting fungi were counted. Relevant references [34,35,36] were consulted for fungal identification to generic/species level. The total count of tested soil microorganisms was recorded as log_10_ colony forming unit (CFU)/10 g soil sample. The fungal frequency percentage was estimated using the following formula:

Frequency of occurrence of a definite fungus (%) = (fungus number/total fungi number) × 100.

### 2.4. Statistical Analysis

Statistical analyses of experimental data were carried out according to ANOVA procedures [37]. Nematode counts were transformed to log_10_ to meet assumptions necessary for parametric statistical analysis. Duncan’s New Multiple Range Test (DMRT) compared means of each microbial set or nematode parameter among the farms in each governorate/sampling time at 5% probability level. Paired *t*-test was used to compare these means recorded after 2 versus 4 months of transplanting for each of the sets/parameters. The *t*-test also compared the overall mean number of RKN parameters between El-Ismailia and El-Beheira, between the two sampling times in each governorate, and between edaphic factors in each of two farms at El-Ismailia and their corresponding overall mean values of the ten remaining farms (Table 3). The detection of factors found in the original data matrix for the fungal frequencies was defined by principal component analysis (PCA) via estimating new variables (principal components) to outline their data distributed in the original variables. The eigenvalues and the eigenvectors generated by PCA are the participation of each constituent to the original variance and their correlation coefficients with the original variables, respectively.

## 3. Results

### 3.1. The Root-Knot Nematodes and Soil Properties

**Figure 1:**
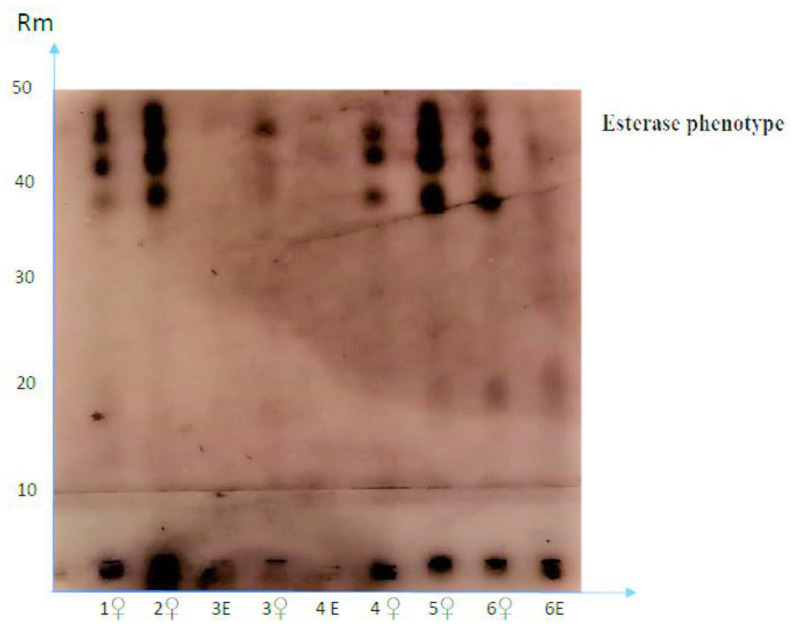
Relative mobility rate (Rm) of esterase isozymes identified from individual root-knot nematode females (♀ and eggs (E) following electrophoresis on polyacrylamide gel. Prevalent esterase phenotypes considered to be specific for *M*. *javanica* are located at the middle of 40 and 50 Rm for the females.

Perineal patterns of 20 *Meloidogyne* females denoted 16 *M*. *javanica*, 3 *M*. *incognita*, and 1 *M*. *arenaria*. Using the esterase enzymes of six individual females revealed the presence of only *M*. *javanica* (Figure 1). Soil textures across the tested farms ranged from sandy loam to loamy sand (Table 1). Pesticide rates were applied as instructed by the Egyptian Ministry of Agriculture [24]. Agricultural practices for the previous crop of strawberry cultivation at the farms, as well as soil properties concerning pH, electrical conductivity (EC), CaCO_3_%, and organic matter, differed but to varying degrees (Table 1). Additionally, production practices for berry cultivation varied among farms (Table 2). As these practices were not quite equal in terms of the techniques used (e.g., levels of soil tilth, covering duration) and materials (e.g., sheet thickness and color, cultivars), differences in the effectivity of soil disinfestation methods probably varied.

In this context, no PPNs could be detected in the Al-Qalyubia farms, probably due to the use of methyl bromide (two farms) or Agrocelhone (four farms) as pre-plant soil fumigants against pests and pathogens. Therefore, no further analysis was conducted there. Soil properties did not significantly differ on a regional scale between the two governorates, except for pH and EC/PPM (Table 3). Although all the surveyed farms possess alkaline soils, those of El-Ismailia showed higher pH but lower electrical conductivity mean values than those for the soils of El-Beheira. Farm-1 and Farm-4 had the same loamy sand texture where each of their three main components (sand, silt, and clay) did not differ significantly from the mean of the ten other farm soils. Each of the two farms had higher pH and lower EC values than the corresponding means of the ten other soils (four of El-Ismailia and six of El-Beheira). The organic matter value was less in Farm-4 than the corresponding mean of these ten soils (Table 3).

The most common PPNs found in soil and root samples of the surveyed farms in El-Ismailia and El-Beheira were *Meloidogyne* nematodes. The genera *Criconemoides*, *Helicotylenchus*, *Tylenchorhynchus*, and *Pratylenchus* were also present in soil, but accounted for 0.8% of the total PPN community. Their numbers were too few to be analyzed. Root-knot nematodes were detected in all the strawberry farms of El-Ismailia and El-Beheira. The population densities of RKN parameters varied significantly (*p* ≤ 0.05) among the six farms at each sampling time in El-Ismailia (Table 4) and El-Beheira (Table 5). Their overall mean in soil and strawberry roots varied from almost zero (or non-detectable level) at either of the sampling times in El-Ismailia to 332 individuals in El-Beheira at the second sampling time. The paired *t*-test revealed that the overall mean value of RKN parameters, i.e., J2 in soil and roots, galls, egg masses, females, and eggs in strawberry roots per plant at the two sampling times, all combined together, was fewer (*p* < 0.0001) in the El-Ismailia farms than its corresponding mean in El-Beheira (mean ± standard error mean was 23 ± 7.48 versus 97 ± 26.33). The difference in the mean value of total RKN parameters between the two sampling times across the six farms was not quite significant (*p* = 0.069, *n* = 6) in El-Ismailia (Table 4) but highly significant (*p* = 0.004, *n* = 6) in El-Beheira (Table 5). Further comparison of values between the two sampling times for each specific RKN life-stage showed a significant increase in nematode juveniles in soil (*p* = 0.030) and roots (*p* = 0.011), galls on roots (*p* = 0.013), and nematode-egg masses (*p* = 0.019) after five months of the growing season in the El-Beheira farms. Such an increase was significant for only two RKN variables, i.e., nematode juveniles in soil (*p* = 0.044) and galls on strawberry roots (*p* = 0.041) in the El-Ismailia farms. Although the values of these RKN parameters increased after five months of the growing season in 10 farms, the other 2 El-Ismailia farms—Farm-1 and Farm-4—contained virtually no nematodes in the second sampling time (Table 4); i.e., no RKNs were detected after five months of the growing season in these two farms. Because nematode-suppressive soils are frequently marked with a decline in PPN population densities after initial establishment, these two farms (Table 4) are of interest to define possible factors and mechanisms that suppressed their RKN population level. The two farms were characterized by incorporating cow manure [containing 0.65% total nitrogen and 21.2 carbon to nitrogen (C/N) ratio] and poultry manure (0.72% total nitrogen, 20.1 C/N ratio) followed by soil solarization via transparent, 80-µm-thick cover sheets for 60–65 summer days as pre-strawberry cultivation practices, and similar sheet thickness and color after transplanting (Table 2). Farm-3 had generally low RKN population densities with fewer RKN eggs (*p* ≤ 0.05) than the other three El-Ismailia farms. Generally, the longer the pre-plant soil solarization duration, the better it could reduce the RKN population densities in the surveyed farms (Table 2, Table 4 and Table 5).

### 3.2. The Microbial Count and Frequency

The overall means of bacterial and fungal counts in El-Ismailia were always higher than those of the El-Beheira farms but varied in magnitudes (Table 6). Apparently, this occurrence is parallel to the above-mentioned lower (*p* > 0.0001) values of RKN parameters in the El-Ismailia farms than their corresponding ones in El-Beheira. In other words, the greater abundance of bacterial and fungal counts in El-Ismailia than the El-Beheira farms and their possible interactions as BCAs with agricultural practices and soil properties (Table 1 and Table 2) were conceivably reflected in the significant reduction in the populations of *Meloidogyne* spp. in El-Ismailia compared to the El-Beheira farms. Using the paired *t*-test, the overall mean of the total microbial (bacterial and fungal) count in rhizosphere soils of the surveyed strawberry farms in El-Ismailia was not quite significantly (*p* = 0.072, *n* = 6) different from its corresponding value in the farms of El-Beheira. This mean ± standard error was 5.032 Log_10_/10g soil ± 0.407 in El-Ismailia versus 4.753 Log_10_/10g soil ± 0.473 in El-Beheira (Table 6). The *t*-test revealed that the mean of spore-forming bacterial counts in the El-Ismailia farms (4.25 Log_10_/10g soil ± 0.159) was significantly (*p* = 0.002, *n* = 6) higher than that in the El-Beheira farms (3.49 Log_10_/10g soil ± 0.099) in the second sampling time. Such a difference was not significant for either the aerobic bacterial (*p* = 0.139, *n* = 6) or the fungal (*p* = 0.642, *n* = 6) counts.

The frequency of occurrence for the fungal genera/species found in the rhizospheres of strawberry after three and five months of transplanting differed in the El-Ismailia farms (Table 7) from the El-Beheira farms (Table 8). Their means decreased after five months of the growing season in the El-Beheira farms concerning *Aspergillus niger* (*p* = 0.030, *n* = 6) and increased for *Penicillium* spp. (*p* = 0.014, *n* = 6) and *Rhizopus* spp. (*p* = 0.020, *n* = 6). Such a decrease was shown in the El-Ismailia farms in *A. niger* (*p* = 0.042, *n* = 6), and an increase in *Aspergillus* spp. (*p* = 0.030, *n* = 6), *Penicillium* spp. (*p* = 0.0006, *n* = 6), and *Rhizopus* spp. (*p* = 0.00001).

**Figure 2:**
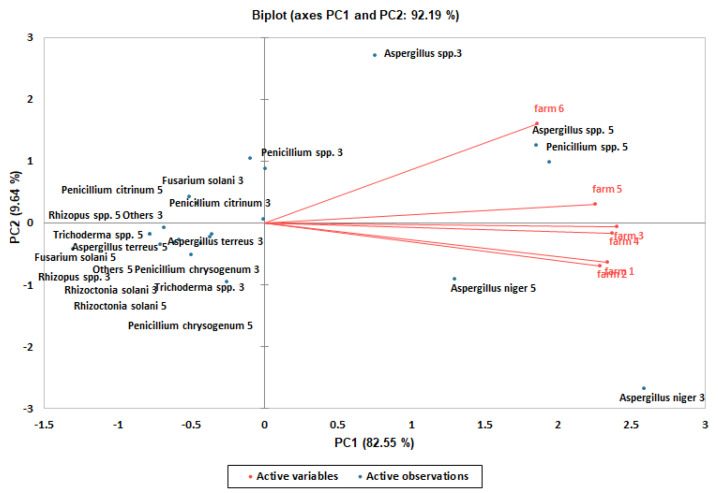
Principal component analysis biplot of first and second principal components of the fungal descriptive profiles in rhizospheres of strawberry in El-Ismailia farms. Farms are shown in red, while fungal groups/species found after 3 and 5 months of transplanting are shown in blue.

**Figure 3:**
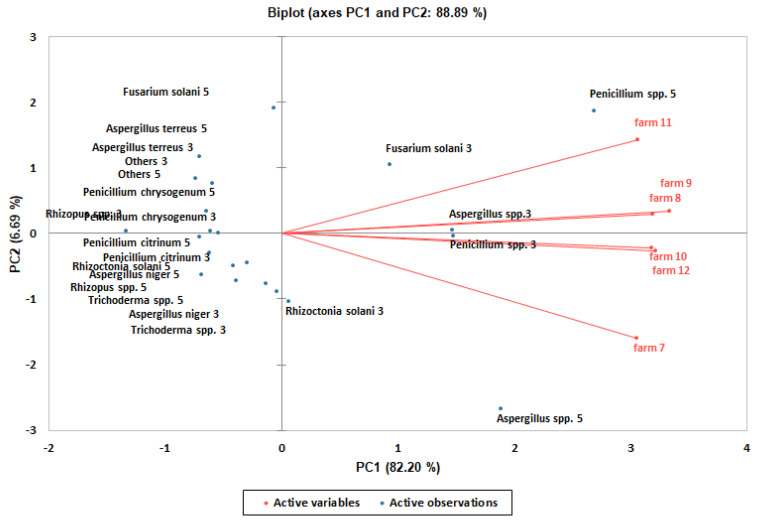
Principal component analysis biplot of first and second principal components of the fungal descriptive profiles in rhizospheres of strawberry in El-Beheira farms. Farms are shown in red, while fungal groups/species found after 3 and 5 months of transplanting are shown in blue.

Using PCA, the variance associated with the first and second principal components was 92.19% of the total in the El-Ismailia farms (Table 9) and 88.89% of the total in the El-Beheira farms (Table 10). Distinct fungal groups/species were identified on the first and second component axes in the El-Ismailia farms (Figure 2) and in the El-Beheira farms (Figure 3). In order to clarify differences in the frequencies of these fungal groups/species among the examined farms, averages of these frequencies were separated using DMRT. Consequently, significant (*p* ≤ 0.05) differences were found among their levels of frequencies in the El-Ismailia farms (Table 7) and in the El-Beheira farms (Table 8).

## 4. Discussion

Soil suppressiveness to nematodes was not previously detected in Egyptian strawberry farms. Yet, biological soil disinfestation and soil suppressiveness have generally been reported in other countries [11,20,21,22,23] with certain tests to define specific soil suppressiveness [38]. Moreover, we aimed to also determine the most effective, economical, and safest nematicidal materials and techniques that can be utilized as benign alternatives to toxic chemicals. Admittedly, MB is banned in many countries due to both ecological pollution and health risks. The use of non-authorized MB should be avoided by farmers [1].

Although many factors contributed differently in lowering RKN population levels in the El-Ismailia farms, a few treatments combined in the emergence of soil biodisinfestation or RKN-suppressive soil in the two El-Ismailia farms. Because biosolarization and soil disinfestation are built on a robust research foundation in organic farming, as well as the physical, chemical, and biological characteristics of disease-suppressive soils [38], various mechanisms in PPN suppression are recorded. The involved mechanisms may include: (1) releasing nematicidal compounds found in soil amendments, (2) producing nematicidal materials, e.g., fatty acids, and ammonia during degradation, (3) boosting and/or introducing antagonistic microorganisms, (4) increasing plant tolerance and resistance, and (5) changing soil physiology to be unfavorable for nematode reproduction [39]. Although nematode control efficacy via soil amendments is not always satisfactory, combining all or more than one of these factors can cause PPN suppression in amended soils [39]. Based on a strong historical research foundation [10,38], several factors apparently boosted these mechanisms in the two above-mentioned farms with RKN-soil suppressiveness. These were manifested by a longer pre-plant soil solarization period that had better suppression of the RKN population densities than those in the other farms with short solarization periods. Likewise, they also involved transparent and thicker plastic films, which have more efficacy in raising soil temperatures and, consequently, in suppressing RKN populations [40,41]. Interestingly, the best reduction in RKNs was reported to occur with transparent sheets relative to the colored ones; red, black, green, and blue [40]. All selected farms used cover sheets, but only the two farms with undetected nematode eggs used transparent ones for pre-plant soil solarization. It is likely that increasing both the period of soil solarization and the thickness of the pre-plant transparent plastic covers in the two farms boosted biodisinfestation compared to the other farms. Such initial factors might help in further detection of soils with nematode suppressiveness [42]. Additionally, in chronological order, before strawberry planting season, strawberry Farm-1 and Farm-4 had undergone two production practices with known favorable effects against RKNs (Table 1). These are abamectin in Farm-1 and decomposed sewage products in Farm-4 (Table 1). Abamectin is distinguished from the other insecticides listed in Table 1 as also being an effective bio-nematicide. Due to its effectiveness, abamectin has been recommended to manage RKNs on several highly susceptible crops in Egypt. It is currently used against severely RKN-attacked Solanaceous crops; on tomatoes [43], peppers [44], potatoes [45], and eggplant [46]. Additionally, sewage sludge is known as an important organic soil amendment used for general PPN control [47]. Biosolarization is equally effective to chemical fumigation of soils as it could significantly suppress soil nematode populations in Spanish strawberry fields [48].

Additionally, the chemical composition (e.g., nitrogen content, C/N ratio) of the applied manures strongly impacts soil suppressiveness against RKNs. The optimum range of C/N ratio that help in enhancing specific biochemical, microbiological, and physical processes with subsequent killing or suppressing plant pathogens is 25–35 [49]. Yet, Howell [50] found that a “diet” with a C:N ratio of 24 is the best. The ratio in the two farms was closer to this range/diet than the ratios recorded in the other farms. In addition to the well-known benefits of mulch (e.g., ameliorating water deficit and salt stress), such effective mulching material in the two farms prevents weed growth as a source of nematode development on them before moving to the strawberry roots. Hence, C:N ratio may be a primary factor for RKN-suppressive soils and should not be underestimated. Apart from the two farms, RKN populations were found in different levels in ten farms (Table 4 and Table 5). Regrettably, if near-zero counts of RKNs remain in any of the surveyed farms, their population densities will surely begin to recover after planting a susceptible crop, as has happened elsewhere [41].

The present study detected significantly higher pH and lower EC values in soils of Farm-1 and Farm-4 than the ten other ones (Table 3). As the two farm soils have a favorable impact against RKNs, their pH and EC have likely contributed to nematode suppression. This is corroborated by other reports. Increasing soil pH to 8.1–9.2 resulted in suppressing *Meloidogyne incognita* J2 and eggs on soybean roots [51]. Populations of *Meloidogyne* nematodes showed the greatest response to nematicidal effects in four cotton fields that were in areas with the lowest EC values [52]. In parallel, the abundance of biocontrol agents was greatest in sites with higher pH, but lower EC and less organic matter, in Egyptian citrus orchards [53]. If so, the lack of organic matter in Farm-4 (Table 3) might have resulted from its decomposition due to the multiplication of beneficial microorganisms on it. Many such microorganisms and/or the released compounds of decomposed matter could play a key role against soil pests such as RKNs [15,22,23]. On the contrary, production practices, especially chemical pesticides generally used in the El-Beheira farms (Table 1), have likely suppressed the population levels of the soil biota (Table 8) and their natural ability to regulate or suppress RKNs. These results agree with the intensively managed strawberry production systems in southern Europe, which significantly reduced the ability of the soil food web to suppress parasitic species [54].

Although edaphic conditions can considerably affect soil microorganisms including RKNs [30,55], this study ruled out some of them in the examined farms. Frequently, RKN reproduction on susceptible plants decreases with increase in salinity [56]. Therefore, the salty water of sites near the Suez Canal coast at El-Ismailia might be assumed to lower (*p* > 0.0001) RKN populations of farms there than those of El-Beheira. However, it is likely that the fresh water of the Nile River branches that irrigate these farms have diluted the salinity [57]. Therefore, the lower levels of RKN populations in the El-Ismailia farms are likely modulated by other mechanisms.

The higher means of bacterial and fungal counts in El-Ismailia than those of the El-Beheira farms (Table 6, Table 7 and Table 8) puts forward potential alternative mechanisms. In this respect, the El-Ismailia farms showed a significant increase in the occurrence of possible biocontrol agents, such as *Aspergillus* spp., *Penicillium* spp., and *Rhizopus* spp. Additionally, somewhat higher (*p* = 0.002) population levels of spore-forming bacteria in the El-Ismailia farms than those in the El-Beheira farms in the second sampling time could put forward some of the above-mentioned mechanistic insights for RKN suppression into their role in enhancing biocontrol efficacy against RKNs. In this respect, Egyptian soil is rich in the spore-forming bacteria that have high effectiveness on *Meloidogyne* nematodes, e.g., *Pasteuria penetrans* [58] and *Bacillus subtilis* [59]. In addition, the abundance of *Aspergillus* spp. was found herein to significantly (*P* = 0.0299) increase in the second sampling time in the El-Ismailia farms. In contrast, such an increase occurred, but to a remarkably lesser, non-significant level (*P* = 0.321) in El-Beheira. Several *Aspergillus* species have been known to reduce nematode development in plant roots and rhizosphere soil, with *A. niger* being the most effective in minimizing the RKN population density [60]. Additionally, seed treatments with *A. niger* alone and in combination with other BCAs could control the root-rot disease complex of chickpea caused by *Meloidogyne incognita* and the fungus *Macrophomina phaseolina* [61,62]. Additionally, *Penicillium* spp. are known to have a highly antagonistic effect on various pathogens, e.g., *p*. *chrysogenum* strain (Snef1216) could inhibit egg hatching and increase the mortality of *M*. *incognita*. The inhibition was boosted by increasing the concentration and exposure time of the fungus filtrate [63]. Added to these BCAs, *Rhizopus* spp. showed a high increase (*p* < 0.00001) in their frequencies at the El-Ismailia farms. Species of *Rhizopus* could parasitize on *M*. *javanica* eggs but are less effective than *Aspergillus* and *Penicillium* [64]. While our study referred to these BCAs during the strawberry-growing season, it should be noted that some *Penicillium*, *Aspergillus,* and *Rhizopus* spp. can cause postharvest strawberry diseases [65]. Further exploration should allocate where these BCAs can properly persist, reproduce, compete, and function with other relevant components in IPM strategies [65]. Due to the aforementioned different modes of action by these BCAs, this exploration should also take into account their possible survival in a saprophytic microbial phase.

Harnessing closely related factors to define BCAs, such as optimal temperature, moisture, pH, and EC, should be earnestly attempted to optimize RKN suppression. Clearly, each biological component has its own optimal requirements for multiplication, infection, or antagonistic activity [9,19,66,67,68]. Adequate sampling [69] and sound tests [11,36,70] are imperative to obtain such related data for effective IPM. Therefore, we recommend testing the effects of BCAs against RKNs in strawberry farms on a case-by-case basis. This will properly shape various but optimized techniques for PPN control based on realistic conditions and variables.

A few related issues may need clarification. Although RKN-gall index is commonly used as an indicator of plant damage [30,71], others [72] used the numbers of RKN-sedentary forms as a more objective and measurable parameter than the frequently used gall index. Further RKN parameters could also be valuable, i.e., the number of RKN eggs in fresh roots, used herein [70]. The eggs can provide precise information on RKN fertility (number of viable eggs produced) which are desperately needed to estimate both the expected burden of RKN recycling on the same strawberry plant and infecting the next (rotated) plant. On the other hand, the plant genotypes and associated soil microbiota are reported [21] to cooperate in suppressing PPNs, but the strawberry cultivars tested herein, dominated by ‘Fertona’ and ‘Festival’, are generally known as being susceptible to RKNs in Egypt [1,6,73]. As there are doubts about the pathogenicity of some *Meloidogyne* species to several strawberry cultivars, the species of RKNs found should also be identified on a case-by-case basis for each farm. Moreover, the introduction of strawberry cultivars that have an incompatible reaction to Egyptian RKN species/races is sorely needed. The strawberry ‘Sweet Ann’ was recently classified as a non-host for *Meloidogyne luci* [74]. Further studies are needed to explore differences in susceptibility between cultivars, BCA species, and population levels, and the effectivity of soil disinfestation methods.

Soil biodisinfestation using cow and poultry manure, followed by soil solarization via transparent, 80-µm-thick cover sheets for 60–65 summer days before and after strawberry transplanting, preceded by abamectin or sewage product in the previous season was herein demonstrated using two RKN-suppressive soils. These production practices, combined with the aforementioned soil properties, have apparently supported more native biocontrol populations at the two farms. However, more extensive studies that determine the co-existing BCA species/strains and their exact interactions with nematodes should be carried out.

## 5. Conclusions

This survey demonstrated that RKN population densities could considerably vary from one governorate/farm to another. We found that optimum soil biodisinfestation could demonstrate RKN-suppressive soils in two farms in the El-Ismailia governorate. It is likely that these production practices combined with the above-mentioned soil properties could back more native biocontrol populations at El-Ismailia than the El-Beheira farms. Consequently, in addition to RKNs-soil suppressiveness at the two farms, significant suppression in nematode population densities was apparent at El-Ismailia compared to the El-Beheira farms. This study could provide the first approximation of key production practices and factors that may collectively contribute to distinguishing and operating soil suppressiveness against RKNs. Further studies are justifiable for a more in-depth examination of the mechanisms responsible for RKN-suppressive soils. Molecular analyses to document the existence of microbial species in the rhizosphere of strawberry plants and their possible infection to one or more RKN-life stages are warranted.

## Figures and Tables

**Table 1:** Soil characteristics in El-Ismailia and El-Beheira governorates, Egypt, and production practices for the previous crop of strawberry cultivation, if any *.

Farm No.	pH	EC_1:2.5_	CaCO_3_%	OM%	Sand:Clay:Silt	Soil Texture	Pre-Strawberry Production Practices
**El-Ismailia Farms**							
Farm-1	8.72	0.266	3	1.02	70.8:17.2:12	Loamy sand	Abamectin for peanut pests then fallow
Farm-2	8.31	0.434	2.4	1.02	72.8:17.2:10	Loamy sand	Methomyl and profenofos for corn pests
Farm-3	8.51	1.663	4	1.29	68.8:19.2:12	Sandy loam	Methomyl and profenofos for corn pests
Farm-4	8.31	0.295	2.5	0.68	70.8:17.2:12	Loamy sand	Decomposed sewage products then fallow
Farm-5	8.40	0.265	3	1.53	72.8:17.2:10	Loamy sand	Methomyl for corn pests
Farm-6	8.29	0.204	3.5	1.36	72.8:15:12	Loamy sand	Abamectin for peanut pests then fallow
**El-Beheira Farms**							
Farm-7	8.18	0.804	2.0	0.34	66.8:17.2:12	Loamy sand	Fertilizers only for peanut
Farm-8	7.9	3.68	4.0	1.02	66.8:19.2:14	Loamy sand	*Trichoderma album* and abamectin for peanut
Farm-9	8.30	1.887	2.0	1.36	68.8:17.2:14	Loamy sand	Methomyl for corn pests then fallow
Farm-10	8.28	0.339	2.5	0.85	72.8:15.2:12	Sandy loam	Penconazole for pepper pests
Farm-11	8.10	0.525	3.5	2.04	72.8:17.2:10	Sandy loam	Fallow due to high salinity
Farm-12	7.58	4.11	6	3.4	50.8:25.2:24	Sandy loam	Fallow due to high salinity

* Only practices/nematicides that may directly affect the strawberry nematodes are listed. Six Al-Qalyubia farms with loamy sand or sandy loam soil textures were excluded from further analyses as no nematodes were present, probably due to soil fumigation with methyl bromide in two farms or Agrocelhone (Dichloropropene + Cloropicrin) in four farms; EC_1:2.5_ measured as dS m^−1^.

**Table 2:** Pre-planting practices and strawberry cultivars for preparing twelve farms equally located in two Egyptian governorates *.

Farm No.	Geographic Coordinates, Plow, Fertilizer, and Irrigation ^+^	Plastic Cover Color/Thickness/Duration and Manure	Strawberry Cultivar
El-Ismailia Governorate			
Farm-1	The coordinates: 30°33′27.8″ N 31°57′38.7″ E. Plow depth: 1st, 25–30 cm; 2nd, 55–60 cm; 3rd, 25 cm. Incorporated 20 m^3^ of decomposed cow manure + 10 m^3^ poultry manure before 3rd plow, then irrigation and plastic cover. After removal of the pre-plant cover, add chemical fertilizers, divide the land into beds, apply the irrigation network.	Pre-plant: transparent 80 µm plastic cover for 60 days. Cow manure (0.65% nitrogen, 21.2 C/N ratio), poultry manure (0.72% nitrogen, 20.1 C/N ratio). Post-plant: transparent 60 µm plastic cover from 4-leaf-stage seedlings until the end of the season.	Savana
Farm-2	The coordinates: 30°33′37.0″ N 32°00′30.8″ E. Same as above.	As above. Pre-plant: blue 32 µm sheet for 15 days. Cow manure (4.7% nitrogen, 5 C/N ratio).	Amega
Farm-3	The coordinates: 30°33′33.1″ N 32°00′30.3″ E. Same as above.	Same as Farm-2. Pre-plant sheet for 15 days.	Festival
Farm-4	The coordinates: 30°33′07.6″ N 32°02′50.0″ E. Same as above.	Same as Farm-1. Cow manure (0.65% nitrogen, 21.2 C/N ratio), poultry manure (0.72% nitrogen, 20.1 C/N ratio). Pre-plant sheet lasted 65 days.	
Farm-5	The coordinates: 30°32′54.4″ N 32°02′28.3″ E. Same as above.	Same as Farm-2, pre-plant sheet for 45 days.	
Farm-6	The coordinates: 30°33′19.1″ N 32°02′09.9″ E. Same as above.	Same as Farm-2, pre-plant sheet for 45 days.	
El-Beheira Governorate			
Farm-7	The coordinates: 30°30′04.0″ N 30°32′12.8″ E. Same as above.	Same as Farm-2 but pre-plant sheet for 7 days.	Fertona
Farm-8	The coordinates: 30°32′27.1″ N 30°32′15.1″ E. Same as above.	Same as Farm-2 but pre-plant sheet for 12 days.	
Farm-9	The coordinates: 30°33′43.4″ N 30°34′54.7″ E. Same as above.	Same as Farm-2 but pre-plant sheet for 12 days.	
Farm-10	The coordinates: 30°33′36.3″ N 30°36′47.2″ E. Same as above.	Same as Farm-2 but pre-plant sheet for 12 days.	
Farm-11	The coordinates: 30°33′5.6″ N 30°41′30.0″ E. Same as above.	Same as Farm-2 but pre-plant sheet for 30 days.	
Farm-12	The coordinates: 30°33′5.1″ N 30°42′43.4″ E Same as above.	Same as Farm-2 but pre-plant sheet for 14 days.	Beauty

* Post-planting plastic covers were also included for comparison with pre-planting ones. Chemical fertilizers in all farms included 250 kg (NH₄)₂SO₄+150 kg CaH_6_O_8_P_2_^+2^ + 150 kg K_2_SO_4_ + 50 kg MgSO₄ + 300 kg sulfur per feddan (4200 m^2^). Total nitrogen content and C/N ratio were provided by the seller. The temperature obtained from the Meteorological Authority ranged from 74–94 °F (average 84 °F) in El-Ismailia and 70–96 °F (average 83 °F) in El-Beheira when pre-planting plastic covers were used. Strawberry cultivars were Fertona (Farms -1, -2 and -5), Festival + Fertona (Farm-3), Sweet Sensation (Farm-4), and Festival + Fertona (Farm-6) in Al-Qalyubia.

**Table 3:** Soil properties in the two governorates of the strawberry farms in Egypt.

Variable	Governorate	Mean ± SE	Min–Max	Statistical Probability Value (*p*)		
				Governmental Differences	Farm-1	Farm-4
Sand %	El-Ismailia	71.47 ± 0.67	68.8–72.8	0.171 ^ns^	0.330 ^ns^	0.330 ^ns^
	El-Beheira	66.47 ± 3.32	50.8–72.8			
Silt %	El-Ismailia	11.33 ± 0.42	10–12	0.178 ^ns^	0.464 ^ns^	0.464 ^ns^
	El-Beheira	14.33 ± 2.03	10–24			
Clay %	El-Ismailia	17.17 ± 0.54	15–19.2	0.393 ^ns^	0.414 ^ns^	0.414 ^ns^
	El-Beheira	18.53 ± 1.43	15.2–25.2			
OM %	El-Ismailia	1.15 ± 0.12	0.68–1.53	0.463 ^ns^	0.159 ^ns^	0.019 **
	El-Beheira	1.50 ± 0.44	0.34–3.4			
Parts per million (ppm)	El-Ismailia	312.83 ± 126.82	131–939	0.074 *	0.038 **	0.042 **
	El-Beheira	1210.17 ± 430.52	217–2630			
pH	El-Ismailia	8.42 ± 0.068	8.29–8.72	0.019 **	0.0001 ***	0.175 ^ns^
	El-Beheira	8.06 ± 0.112	7.58–8.30			
CaCO_3_	El-Ismailia	3.08 ± 0.302	2.4–4	0.742 ^ns^	0.472 ^ns^	0.070 *
	El-Beheira	3.33 ± 0.628	2–6			

Notes: Mean ± standard error mean, maximum and minimum values, and the significant regional differences are represented for each component (df = 10). Farms -1 and -4 indicate whether each of the two farms represents significant difference from the other ten farms for each component (df = 9). *p < 0.10; **p < 0.05; ***p < 0.01; ^ns^ = non-significant; ppm = mg of salts/l of water.

**Table 4:** Population density of root-knot nematode parameters in six strawberry farms of El-Ismailia governorate, Egypt, after three and five months of transplanting *.

Second-Stage Juveniles in Soil				Second-Stage Juveniles in Roots		Galls on Roots		Egg-Masses in Roots		Females in Roots		Eggs in Roots		Total RKN Count (200 g Soil + 5 g Roots)	
	**Months**	**3**	**5**	**3**	**5**	**3**	**5**	**3**	**5**	3	**5**	3	**5**	3	**5**
**Farm** **No.**															
Farm-1		0 ^b^	0 ^e^	0 ^e^	0 ^d^	0	0 ^e^	0	0 ^c^	5 ^c^	0 ^d^	17 ^c^	0 ^e^	22	0
Farm-2		0 ^b^	119 ^a^	268 ^a^	150 ^a^	0	5 ^a^	0	2 ^a^	19 ^a^	49 ^a^	43 ^a^	140 ^a^	330	465
Farm-3		11 ^a^	49 ^d^	15 ^d^	39 ^c^	0	1 ^d^	0	0 ^c^	14 ^b^	9 ^c^	29 ^b^	38 ^d^	69	136
Farm-4		0 ^b^	0 ^e^	49 ^b^	0 ^d^	0	0 ^e^	0	0 ^c^	3 ^c^	0 ^d^	14 ^c^	0 ^e^	66	0
Farm-5		0 ^b^	78 ^b^	0 ^e^	49 ^c^	0	4 ^b^	0	1 ^b^	0 ^d^	39 ^b^	0 ^d^	78 ^c^	0	249
Farm-6		0 ^b^	59 ^c^	40 ^c^	70 ^b^	0	2 ^c^	0	1 ^b^	4 ^c^	49 ^a^	14 ^c^	99 ^b^	58	280
Mean		2	51	62	51	0	2	0	1	8	24	20	59	91	188

* Means in each column followed by the same small letter are not significantly (*p* ≤ 0.05) different according to Duncan’s New Multiple Range Test. All fractional values rounded up to nearest integer.

**Table 5:** Population density of root-knot nematode parameters in six strawberry farms of El-Beheira governorate, Egypt, after three and five months of transplanting *.

Second-Stage Juveniles in Soil				Second-Stage Juveniles in Roots		Galls on Roots		Egg-Masses in Roots		Females in Roots		Eggs in Roots		Total RKN Count (200 g Soil + 5 g Roots)	
	**Months**	**3**	**5**	**3**	**5**	**3**	**5**	**3**	**5**	3	**5**	3	**5**	3	**5**
**Farm** **No.**															
Farm-7		346 ^a^	524 ^a^	245 ^a^	911^a^	2 ^b^	9 ^b^	1 ^b^	6 ^a^	34 ^a^	181^a^	218 ^a^	362 ^a^	846	1993
Farm-8		140 ^b^	185 ^b^	109 ^b^	416^c^	3 ^b^	6 ^c^	1 ^b^	4 ^b^	19 ^b^	99 ^c^	106 ^b^	199 ^c^	378	909
Farm-9		42 ^d^	78 ^d^	59 ^c^	281 ^d^	14 ^a^	13 ^a^	5 ^a^	7 ^a^	15 ^b^	150^b^	119 ^b^	301 ^b^	254	830
Farm-10		59 ^c^	119 ^c^	49 ^d^	500 ^b^	2 ^b^	5 ^c^	1^b^	3 ^b^	9 ^bc^	59 ^d^	70 ^c^	119 ^d^	190	805
Farm-11		9 ^f^	49 ^e^	0 ^f^	99 ^f^	0 ^c^	2 ^d^	0 ^c^	1 ^c^	0 ^c^	19 ^f^	0 ^e^	49 ^e^	9	217
Farm-12		19 ^e^	208 ^b^	29 ^e^	125 ^e^	2 ^b^	5 ^c^	1^b^	7 ^a^	7 ^bc^	49 ^e^	35 ^d^	49 ^e^	93	443
Mean		103	194	82	389	4	7	2	5	14	93	91	180	295	866

* Means in each column followed by the same small letter are not significantly (*p* ≤ 0.05) different according to Duncan’s New Multiple Range Test. All fractional values rounded up to nearest integer.

**Table 6:** Averages of total microbial count of spore-forming bacteria, aerobic bacteria, and fungi in rhizosphere soils of strawberry farms in El-Ismailia and El-Beheira governorates, Egypt, after three and five months of transplanting *.

FARM NO	Microbial Count Log_10_/10 g Soil					
	Spore-Forming Bacteria		Aerobic Bacteria		Fungi	
	**3**	**5**	**3**	**5**	**3**	**5**
El-Ismailia farms						
1	4.23 ^bc^	3.65 ^d^	6.86 ^b^	6.24 ^d^	4.34 ^b^	4.43 ^b^
2	3.78 ^d^	3.94 ^c^	6.95 ^a^	6.40 ^b^	4.58 ^a^	4.48 ^b^
3	3.98 ^cd^	4.27 ^b^	6.53 ^d^	6.10 ^e^	4.57 ^a^	4.24 ^c^
4	4.92 ^a^	4.59 ^a^	6.65 ^c^	6.33 ^c^	4.54 ^a^	4.78 ^a^
5	4.41 ^b^	4.66 ^a^	5.52 ^e^	6.49 ^a^	4.52 ^a^	4.81 ^a^
6	4.42 ^b^	4.40 ^b^	5.22 ^f^	6.42 ^b^	4.53 ^a^	4.40 ^b^
Mean	4.29	4.25	6.29	6.33	4.51	4.52
El-Beheira farms						
7	3.86 ^b^	3.55 ^b^	6.37 ^c^	6.12 ^c^	4.47 ^b^	4.57 ^a^
8	4.03 ^ab^	3.40 ^b^	6.55 ^a^	6.18 ^b^	4.26 ^c^	4.52 ^a^
9	3.46 ^c^	3.16 ^c^	5.90 ^d^	6.29 ^a^	4.39 ^b^	4.31 ^c^
10	2.49 ^d^	3.89 ^a^	5.72 ^e^	6.31 ^a^	4.43 ^b^	4.56 ^a^
11	4.32 ^a^	3.55 ^b^	5.53 ^f^	6.27 ^a^	4.61 ^a^	4.40 ^b^
12	4.39 ^a^	3.40 ^b^	6.46 ^b^	6.18 ^b^	4.65 ^a^	4.49 ^a^
Mean	3.76	3.49	6.09	6.23	4.47	4.48

* Means in each column followed by the same small letter are not significantly (*p* ≤ 0.05) different according to Duncan’s New Multiple Range Test.

**Table 7:** The frequency % of fungi in rhizospheres of strawberry in El-Ismailia farms after 3 and 5 months of transplanting under field conditions *.

Common Fungi		*Aspergillus niger*		*Aspergillus terreus*		*Aspergillus* spp.		*Penicillium citrinum*		*Penicillium chrysogenum*		*Penicillium* spp.		*Trichoderma* spp.		*Fusarium solani*		*Rhizoctonia solani*		*Rhizopus* spp.		Others	
**Sowing** **Months**		**3**	**5**	**3**	**5**	**3**	**5**	**3**	**5**	**3**	**5**	**3**	**5**	3	**5**	3	**5**	3	**5**	3	**5**	3	**5**
Frequency % of fungi at farm No.	1	35.7 ^a^	25.8 ^A^	7.1 ^b^	3.2 ^B^	10.7 ^bc^	19.4 ^C^	14.3 ^a^	3.2 ^B^	7.1 ^b^	6.5 ^B^	7.1 ^b^	22.6 ^B^	7.1 ^ab^	3.2 ^B^	3.6 ^b^	6.5 ^A^	3.6 ^b^	3.2	0.0	3.2	3.7	3.2
	2	33.3 ^a^	24.1 ^A^	6.7 ^b^	3.5 ^B^	10.0 ^c^	17.2 ^D^	3.3 ^c^	3.5 ^B^	3.3 ^c^	6.9 ^B^	6.7 ^b^	24.1 ^AB^	13.3 ^a^	3.5 ^B^	6.7 ^b^	3.5 ^B^	10.0 ^a^	3.5	0.0	3.5	6.7	6.7
	3	29.2 ^a^	20.0 ^B^	4.2 ^bc^	8.0 ^A^	12.5 ^b^	24.0 ^A^	12.5 ^a^	4.0 ^B^	4.2 ^c^	4.0 ^C^	12.5 ^a^	20.0 ^B^	4.2 ^b^	4.0 ^B^	8.3 ^ab^	4.0 ^B^	4.2 ^b^	4.0	0.0	4.0	8.2	4.0
	4	28.6 ^a^	14.8 ^C^	3.6 ^c^	7.4 ^A^	10.7 ^bc^	22.2 ^B^	7.1 ^b^	7.4 ^A^	10.7 ^a^	3.7 ^C^	7.1 ^b^	25.9 ^A^	10.7 ^a^	3.7 ^B^	10.3 ^a^	3.7 ^B^	7.1 ^a^	3.7	0.0	3.7	4.1	3.8
	5	25.0 ^a^	10.7 ^C^	12.5 ^a^	3.6 ^B^	16.7 ^b^	25.0 ^A^	8.3 ^b^	7.1 ^A^	8.3 ^b^	10.7 ^A^	8.3 ^b^	17.9 ^B^	4.2 ^b^	7.1 ^A^	8.3 ^ab^	3.6 ^B^	4.2 ^b^	3.6	0.0	3.6	4.2	7.1
	6	9.1 ^b^	15.4 ^C^	4.6 ^bc^	3.9 ^B^	27.3 ^a^	23.1 ^AB^	9.1 ^b^	7.7 ^A^	4.6 ^c^	3.9 ^C^	13.6 ^a^	26.9 ^A^	4.6 ^b^	3.9 ^B^	13.6 ^a^	3.9 ^B^	4.6 ^b^	3.9	0.0	3.9	8.9	3.5
Total mean		26.8	18.5	6.5	4.9	14.7	21.8	9.1	5.5	6.4	6	9.2	22.9	7.4	4.2	8.5	4.2	5.6	3.7	0	3.7	6	4.7

* Means in each column followed by the same letter are not significantly (*p* ≤ 0.05) different according to Duncan’s New Multiple Range Test.

**Table 8:** The frequency % of fungi in rhizospheres of strawberry in El-Beheira farms after 3 and 5 months of transplanting under field conditions *.

Common Fungi		*Aspergillus niger*		*Aspergillus terreus*		*Aspergillus* spp.		*Penicillium citrinum*		*Penicillium chrysogenum*		*Penicillium* spp.		*Trichoderma* spp.		*Fusarium solani*		*Rhizoctonia solani*		*Rhizopus* spp.		Others	
**Sowing** **Months**		**3**	**5**	**3**	**5**	**3**	**5**	**3**	**5**	**3**	**5**	**3**	**5**	3	**5**	3	**5**	3	**5**	3	**5**	3	**5**
Frequency % of fungi at farm No.	7	11.1 ^b^	8.3 ^B^	0.0 ^c^	0.0 ^C^	16.7 ^b^	29.2 ^A^	5.6 ^b^	4.2 ^B^	5.6 ^b^	4.2 ^B^	17.2 ^ab^	20.8 ^B^	12.5 ^a^	8.6 ^A^	11.1 ^b^	4.2 ^B^	16.7 ^a^	7.1 ^A^	0.0	8.3 ^A^	3.5 ^b^	5.1 ^AB^
	8	13.3 ^a^	11.5 ^A^	0.0 ^c^	3.9 ^B^	20.0 ^a^	19.2 ^C^	3.3 ^b^	3.9 ^B^	10.0 ^a^	7.7 ^A^	20.0 ^a^	26.9 ^A^	3.9 ^c^	3.3 ^C^	20.0 ^a^	7.7 ^AB^	3.3 ^b^	7.7 ^A^	0.0	3.9 ^B^	6.2 ^a^	4.3 ^BC^
	9	8.0 ^c^	3.5 ^C^	12.0 ^a^	3.5 ^B^	20.0 ^a^	24.1 ^B^	4.0 ^b^	3.5 ^B^	4.0 ^b^	3.5 ^B^	20.0 ^a^	31.0 ^A^	8.0 ^b^	8.0 ^A^	16.0 ^ab^	10.4 ^A^	4.0 ^b^	6.9 ^AB^	0.0	3.5 ^B^	4.0 ^b^	2.1 ^C^
	10	8.3 ^c^	6.5 ^B^	4.2 ^b^	3.2 ^B^	20.8 ^a^	19.4 ^C^	8.3 ^a^	6.5 ^A^	4.2 ^b^	3.2 ^B^	20.8 ^a^	32.3 ^A^	12.5 ^a^	6.5 ^B^	4.2^c^	6.5 ^AB^	12.5 ^a^	9.7 ^A^	0.0	3.2 ^B^	4.2 ^b^	3.0 ^BC^
	11	4.2 ^d^	4.0 ^C^	4.3 ^b^	8.0 ^A^	16.7 ^b^	12.0 ^D^	4.2 ^b^	4.0 ^B^	4.4^b^	4.0 ^B^	16.7 ^b^	32.0 ^A^	8.0 ^b^	4.2 ^C^	16.7^ab^	16.0 ^A^	12.5 ^a^	4.0 ^B^	0.0	4.0 ^B^	8.1 ^a^	7.8 ^A^
	12	4.0 ^d^	3.5 ^C^	4.0 ^b^	6.9 ^A^	20.0 ^a^	27.6 ^A^	4.0 ^b^	3.5 ^B^	4.0^b^	6.9 ^A^	20.0 ^a^	20.7 ^B^	8.0 ^b^	8.0 ^A^	24.0^a^	6.9 ^AB^	8.0 ^ab^	6.9 ^AB^	0.0	3.5 ^B^	4.0 ^b^	5.6 ^AB^
Total mean		8.2	6.2	4.1	4.3	19	21.9	4.9	4.3	5.4	4.9	19.1	27.3	8.8	6.4	15.3	8.6	9.5	7.1	0.0	4.4	5	4.7

* Means in each column followed by the same letter are not significantly (*p* ≤ 0.05) different according to Duncan’s New Multiple Range Test.

**Table 9:** Eigenvalues of the correlation matrix used for principal component analysis of frequency % for various fungi in rhizospheres of strawberry in El-Ismailia farms after 3 and 5 months of transplanting and proportion and cumulative percentages of total variance explained by each principal component.

Principal Component	Eigenvalue	Proportion	Cumulative
First	4.953	82.553	82.553
Second	0.578	9.641	92.194
Third	0.210	3.499	95.694

**Table 10:** Eigenvalues of the correlation matrix used for principal component analysis of frequency % for various fungi in rhizospheres of strawberry in El-Beheira farms after 3 and 5 months of transplanting and proportion and cumulative percentages of total variance explained by each principal component.

Principal Component	Eigenvalue	Proportion	Cumulative
First	4.932	82.196	82.196
Second	0.402	6.694	88.890
Third	0.324	5.403	94.293

## Data Availability

Not applicable.
